# University entrance exam anxiety of adolescents during COVID-19 pandemic: Cognitive flexibility and self-regulation

**DOI:** 10.1192/j.eurpsy.2021.1771

**Published:** 2021-08-13

**Authors:** M. Korhan, E. Engin, B. Güloğlu

**Affiliations:** 1 School Counseling, Bahcesehir Colleges, Tekirdag, Turkey; 2 No Department, Independent Researcher, İstanbul, Turkey; 3 Psychological Counseling, Bahcesehir University, Istanbul, Turkey

**Keywords:** test anxiety, COVID-19, self-regulation, cognitive flexibility

## Abstract

**Introduction:**

After the first case of Covid-19 was emerged in Turkey on March 11, 2020, schools were closed distance education began. On March 21, curfew was declared for people who under the age of 20. Subsequently, the date of the nationwide university exam was changed twice.

**Objectives:**

This study has two aims. The first goal was to investigate the factors that affect the test anxiety of individuals who will enter the university exam during the Covid-19 outbreak. The second aim was to investigate whether the test anxiety levels of the participants vary according to the level of cognitive flexibility and self-regulation.

**Methods:**

The study consists of 420 (284 women, 131 men and 5 others). The age range of the participants was between 18 and 21, with the mean of 18.33. Exam Anxiety Scale, Adolescent Self-Regulation Skills Scale and Cognitive Flexibility Scale were used to collect data.

**Results:**

The findings indicated that women’s level of test anxiety was higher than men. Those who do have concentration issues in distance education have high level of test anxiety. Test anxiety was higher for students whose household income decreased because of the pandemic. MANOVA results revealed that individuals with low test anxiety have higher ‘self-regulation successful’ scores and lower ‘self-regulation unsuccessful’ scores. Moreover, it was yielded that people with low test anxiety have higher level of cognitive flexibility.

**Conclusions:**

Protective factors such as cognitive flexibility and self-regulation play an important role in individuals’ management of test anxiety.

**Disclosure:**

No significant relationships.

